# Implementation of model-informed precision dosing for tamoxifen therapy in patients with breast cancer: A prospective intervention study

**DOI:** 10.1016/j.breast.2025.103880

**Published:** 2025-01-09

**Authors:** Ruben Y.M. van Nijnatten, Sanne M. Buijs, Bram C. Agema, Raphaël M.J. Fischer, Inge Ghobadi Moghaddam-Helmantel, Caroline M.E. Contant, Felix E. de Jongh, Auke M.T. Huijben, Manon Kop, Annemieke van der Padt-Pruijsten, Hanneke J.M. Zuetenhorst, Ron H.N. van Schaik, Birgit C.P. Koch, A. Jager, Stijn L.W. Koolen, Ron H.J. Mathijssen

**Affiliations:** a Department of Medical Oncology, Erasmus MC Cancer Institute, Rotterdam, the Netherlands; b Department of Surgery, Maasstad Hospital, Rotterdam, the Netherlands; c Department of Internal Medicine, Breast Cancer Center South Holland South, Ikazia Hospital, Rotterdam, the Netherlands; d Department of Internal Medicine, Breast Cancer Center South Holland South, Maasstad Hospital, Rotterdam, the Netherlands; e Department of Internal Medicine, IJsselland Hospital, Capelle aan den IJssel, the Netherlands; f Department of Internal Medicine, Breast Cancer Center South Holland South, Spijkenisse Medical Center, Spijkenisse, the Netherlands; g Department of Internal Medicine, Franciscus Gasthuis & Vlietland, Schiedam, the Netherlands; h Department of Clinical Chemistry, Erasmus University Medical Center, Rotterdam, the Netherlands; i Department of Hospital Pharmacy, Erasmus University Medical Center, Rotterdam, the Netherlands

**Keywords:** Breast cancer, Estrogen receptor positive, Hormone therapy, Tamoxifen, Endoxifen, Model informed precision dosing, Therapeutic drug monitoring

## Abstract

Tamoxifen is an estrogen-receptor (ER) antagonist, used as adjuvant treatment of ER-positive breast cancer. It is converted by CYP2D6 into endoxifen, its most active metabolite. Patients with endoxifen plasma concentrations <16 nM face a higher risk of recurrence. The use of *a priori* model-informed precision dosing (MIPD) may lead to faster target attainment and thus potentially improve patient outcomes.

In total, 106 evaluable patients were prospectively included in this single-arm MIPD-intervention study. Patients received a model-predicted tamoxifen dose when starting tamoxifen-treatment (65.1 % of patients received 20 mg, 16.0 % received 30 mg and 18.9 % received 40 mg). Seventy-five percent of the 40 mg group was predicted to be unable to reach the threshold of 16 nM despite receiving the highest registered dose. After attaining steady-state, 84.0 % of patients reached endoxifen levels ≥16 nM, which was not significantly higher compared to a historical control cohort (77.9 %, *p* = 0.17). The model showed adequate performance and correctly identified patients requiring 40 mg tamoxifen. Endoxifen samples that were acquired 4–6 weeks after treatment initiation, are informative of steady-state endoxifen levels and can be used to inform MIPD and adjust tamoxifen dosing prior to steady-state attainment.

In this first MIPD implementation study for patients treated with tamoxifen, MIPD did lead to more patients achieving endoxifen levels ≥16 nM as compared to the one-dose-fits-all strategy, albeit insignificant. This may partly be explained by a larger proportion of patients who were recommended to switch to an aromatase inhibitor (AI) in the intervention cohort. In conclusion, MIPD seems beneficial compared to one-size-fits-all-dosing, but TDM still remains an important addition.

## Highlights

• Providing the adequate tamoxifen dose for adjuvant treatment may reduce recurrence.
• Using model-informed dosing a personalized dose can be identified prior to therapy.
• Implementation led to more patients achieving endoxifen ≥16 nM, albeit insignificant.
• The model correctly identified patients unable to reach endoxifen ≥16 nM.
• Although model-informed dosing seems beneficial TDM remains an important addition.

## 1 Introduction

Tamoxifen is a selective estrogen-receptor (ER) modulator and is important in the adjuvant treatment of ER-positive breast cancer where it reduces the breast cancer recurrence rate [1,2]. Tamoxifen is a prodrug and exerts its effect primarily through its active metabolite endoxifen [3,4]. Tamoxifen is converted into endoxifen by cytochrome P450 (CYP) iso-enzymes, particularly by CYP2D6. Polymorphisms of the *CYP2D6* gene can hamper CYP2D6 activity and lead to lower endoxifen concentrations [5,6]. Madlensky et al. found an association between endoxifen concentrations and breast cancer recurrence in a large retrospective analysis of a prospective study [7]. Patients with endoxifen concentrations <16 nmol/L (5.97 ng/mL) were exposed to a 30 % higher risk of breast cancer recurrence compared to patients with endoxifen concentrations above this threshold [7].

Approximately 20–24 % of tamoxifen patients do not reach the 16 nmol/L endoxifen threshold while treated with the standard tamoxifen dose of 20 mg [[7], [8], [9], [10]]. When applying Therapeutic Drug Monitoring (TDM), doses are adapted to measured plasma concentrations to reach the therapeutic threshold. In past studies, implementation of TDM resulted in approximately 90 % of patients with endoxifen levels above the 16 nM threshold after 6 months of tamoxifen therapy [[8], [9], [10], [11]]. One drawback of TDM of tamoxifen in its current form, however, is that the dose adjustments can only be performed after reaching steady-state plasma concentrations, which for tamoxifen is reached after 3 months of therapy [12]. Consequently, patients requiring a tamoxifen dose adjustment after TDM are potentially undertreated during the first 3–6 months of therapy. Model-informed precision dosing (MIPD) may counter this problem by both predicting the adequate tamoxifen dose per patient before the start of treatment and identifying patients who will not reach the 16 nM threshold despite using the highest registered dose and therefore may profit from a switch toward an aromatase inhibitor (AI). Although it is under debate on what falls within the term of MIPD, we refer to using a population-pharmacokinetic (popPK) model, capable of describing and predicting patient-specific absorption, distribution, metabolism, and elimination of a drug based on several patient characteristics, to forecast plasma concentrations before treatment [13]. However, it has never been prospectively investigated whether MIPD for tamoxifen leads to an increase in the proportion of patients inside the therapeutic interval while avoiding unnecessary dose increases. Therefore, the primary aim of this study was to investigate whether implementation of MIPD could increase the proportion of patients achieving an endoxifen level >16 nM at steady-state.

## 2 Materials and methods

### 2.1 Study type and population

The PREDICTAM (PREDICtion of TAMoxifen) trial was conducted as an open-label, single-arm intervention study at the Erasmus MC Cancer Institute in Rotterdam, the Netherlands. It received approval from the institutional review board (MEC-2022-0437) and was registered in the U.S. National Library of Medicine (clinicaltrials.gov; NCT05525481). Patients were eligible for inclusion when starting adjuvant treatment with tamoxifen for primary breast cancer who were able and willing to abstain from moderate and strong CYP2D6 and CYP3A4 inhibitors. Exclusion criteria were ongoing tamoxifen treatment exceeding two weeks prior to the moment of inclusion (i.e. baseline visit), previous quantification of endoxifen levels and the male sex. The control arm was a cohort derived from the TOTAM-study (MEC-2017-548), with which the popPK model was developed [14]. This cohort was followed-up prospectively at the Erasmus MC Cancer Institute using the same catchment area and in- and exclusion criteria as the interventional arm. It consisted of 443 patients, all treated with the standard dose of 20 mg tamoxifen during the first 3 months, of whom 345 (77.9 %) reached the endoxifen threshold of 16 nM at 3 months of treatment (steady-state) [14]. Randomization was not performed in this study as the intervention arm consisted of the entire PREDICTAM cohort and the control arm of the entire cohort derived from the TOTAM study, which rendered the study an open-label study.

### 2.2 Study design

This study used a previously described validated popPK model to predict a patient's steady-state endoxifen plasma concentration and determine the appropriate tamoxifen dose at baseline [14]. Details regarding the prediction model and for which patient each dose was predicted can also be found in the **Supplementary (Section I).** The study consisted of three visits: at baseline (before or within 2 weeks after start of tamoxifen), after 4–6 weeks of tamoxifen therapy, and after 3 months of tamoxifen therapy or, when performed, after a dose-escalation (i.e. steady-state). At baseline, informed consent was obtained and all patient covariates needed for dose prediction were collected (CYP2D6 genotype, body mass index (BMI), age, and body height. Also, patients were asked to fill in the Functional Assessment of Cancer Therapy – Endocrine Symptoms (FACT-ES) questionnaire regarding endocrine side effects and health-related quality of life (HR-QOL). In case patients had already started tamoxifen treatment, an endoxifen plasma sample was collected. After determination of the CYP2D6 genotype **(Supplementary** **Section II)**, a model-informed tamoxifen dose of either 20, 30, or 40 mg was determined and prescribed. During the second visit, after 4–6 weeks of treatment, another plasma sample was taken to investigate whether pre-steady-state endoxifen plasma samples could be indicative of steady-state endoxifen levels. As the early plasma samples were analyzed after completion of the trial, this data did not influence the advised dose during the study. At the last visit, steady-state endoxifen concentrations **(Supplementary** **Section II)** were measured and patients filled in the FACT-ES questionnaire for the second time.

### 2.3 Statistical analysis

The primary endpoint of this study was the proportion of patients with an endoxifen level ≥16 nM at steady-state, *i.e.* 3 months after start of therapy or dose-escalation using MIPD. It was hypothesized that the proportion of patients with endoxifen levels ≥16 nM would be at least 90 % and, therefore, higher than the 78 % observed in the historical cohort. To test this hypothesis, with a two-sided alpha of 5 % and a power of 80 %, at least 106 patients were needed in this new cohort. Comparison between the proportions of the historical and new cohorts was conducted using a chi-squared test. Also, the proportions of patients with endoxifen levels ≥16 nM in the different dosing categories were compared with the historical cohort using a chi-squared test or Fisher's exact test when appropriate. All secondary endpoints and corresponding statistical methods can be found in the **Supplementary (Section III**).

## 3 Results

### 3.1 Patient selection

**Fig. 1:**
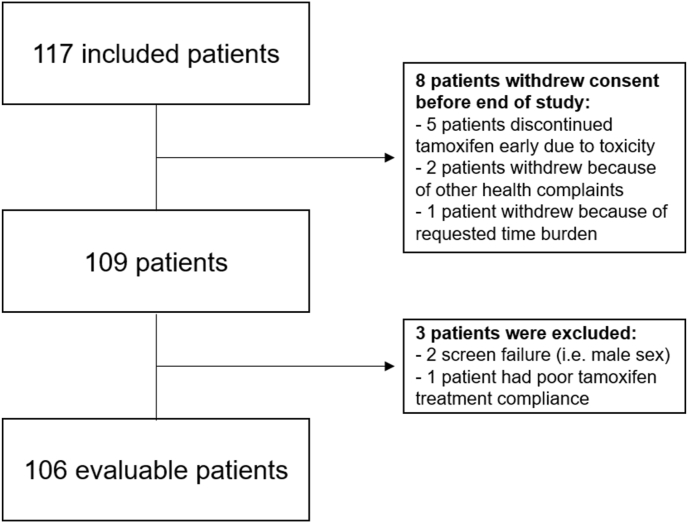
Flowchart for patient selection.

Between November 2022 and September 2023, 117 patients with breast cancer who received or were about to receive adjuvant tamoxifen treatment were enrolled in this study. Eight participants withdrew their consent before reaching the primary endpoint. Five patients withdrew due to experiencing tamoxifen-related side-effects, two patients because of other health complications and one patient because of the requested time burden. Three participants were excluded from the analysis. One participant was excluded due to documented and self-reported poor tamoxifen treatment compliance. Two other patients were excluded before analysis, due to a screening failure (i.e. male sex). Finally, 106 patients were evaluable for the analysis of the primary endpoint (Fig. 1).

### 3.2 Baseline characteristics control and intervention cohort

An overview of baseline characteristics of the control and intervention cohort is provided in Table 1. Patient height and age differed significantly, although not clinically relevant between the intervention and control cohort. For comparison purposes, it was retrospectively determined which model-informed doses would have been predicted in the control group at baseline. Remarkably, significantly more patients in the intervention cohort were predicted in need for an AI, because they would not achieve endoxifen levels >16 nM with tamoxifen while treated with a tamoxifen dose of 40 mg, in the intervention cohort compared to the control cohort. Also, a higher percentage of patients in the intervention group had CYP2D6 activity scores below 0.6, although this difference did not meet significance due to small numbers.

**Table 1:** Baseline characteristics of the evaluable intervention and control patients. All values, except for the predicted dose and CYP2D6 activity groups, were given as medians[IQR]. The combination of two patient alleles was scored using a continuous CYP2D6 activity scale, with 1.0 being a fully active ∗1/∗1 genotype. The predicted dose and CYP2D6 activity groups were given as n(%).

	Intervention cohort (*n* = 106)		Control cohort (*n* = 443)		p-value^c^
Age, *years*	60	[50, 68]	57	[48, 66]	0.049
Height, *cm*	166	[161, 170]	168	[163, 173]	0.001
Weight, *kg*	74	[68, 84]	75	[66, 84]	0.831
BMI, *kg·m^−2^*	27.3	[24.0, 31.3]	26.2	[23.0, 30.0]	0.087
CYP2D6 activity	0.68	[0.48, 0.86]	0.68	[0.51, 0.86]	0.325
Predicted dose					
20 mg	69	(65.0 %)	326^a^	(74.0 %)	0.080
30 mg	17	(16.0 %)	59^a^	(13.0 %)	0.467
40 mg	5	(4.7 %)	24^a^	(5.4 %)	0.772
*(potential) switch to AI*^b^	15	(14.0 %)	34^a^	(7.7 %)	0.036
CYP2D6 activity					
*0.0 – 0.30*	21	(19.8 %)	62	(14.0 %)	0.133
*0.30 – 0.60*	23	(21.6 %)	83	(18.7 %)	0.488
*0.60 – 0.75*	25	(23.6 %)	152	(34.3 %)	0.034
*0.75 – 0.90*	19	(17.9 %)	90	(20.3 %)	0.579
*0.90 – 1.5*	18	(17.0 %)	56	(12.6 %)	0.240

a The predicted dose at baseline in the control group was retrospectively determined for comparison purposes.

b This group was treated with 40 mg to evaluate the chance of reaching endoxifen 16 nM.

c t-test for continuous variables and chi-squared test for distributions of categorical variables.

### 3.3 Endoxifen threshold

Of the 106 patients undergoing MIPD, 89 patients (84 %) reached an endoxifen level ≥16 nM at steady-state. This was not significantly higher compared to the control group (78 %, X^2^ = 1.91; *p* = 0.167). The distribution of steady-state endoxifen levels is depicted in Fig. 1. If patients who were predicted to not reach 16 nM, despite receiving 40 mg, were actually switched to an AI, 91.5 % of the population would have reached endoxifen levels ≥16 nM. The incidence of endoxifen levels ≤16 nM was also stratified per dose group (Table 2). This showed that the proportion of patients with endoxifen levels <16 nM was significantly reduced in the 40 mg and switch to AI groups in the intervention cohort, compared to the dose groups in the control group where no MIPD was performed (Table 2). A list with characteristics of each individual patient with subtherapeutic endoxifen levels <16 nM in the intervention cohort, was given in Supplementary Table 1. Notably, in the 20 mg category, four patients had endoxifen levels <16 nM, despite having favorable CYP2D6 genotypes (*∗1/∗1, ∗1/∗1, ∗1/∗2* and *∗1/∗9,* all corresponding with a CYP2D6 activity >0.75).

**Table 2:** Patients with an endoxifen concentration of <16 nM in the control and intervention cohorts. All patients in the control cohort received 20 mg while in the intervention cohort patients received the predicted dose.

	Patients with endoxifen <16 nM				*p-value*
	Intervention cohort (MIPD)		Control cohort (20 mg)		
	*n*	*%/cohort*^a^	*n*	*%/cohort*^a^	
Predicted dose					
20 mg	5	(7.2 %)	17^a^	(5.2 %)	0.504^b^
30 mg	4	(23.5 %)	26^a^	(44.1 %)	0.164^c^
40 mg	0	(0 %)	21^a^	(87.5 %)	<0.001^c^
*(potential) switch to AI*	8	(53.3 %)	34^a^	(100 %)	<0.001^b^
CYP2D6 activity					
*0.0 – 0.30*	9	(42.9 %)	59	(95.2 %)	<0.001^b^
*0.30 – 0.60*	4	(17.4 %)	25	(30.1 %)	0.295^c^
*0.60 – 0.75*	0	(0.0 %)	10	(6.6 %)	0.361^c^
*0.75 – 0.90*	3	(15.8 %)	4	(4.4 %)	0.100^c^
*0.90 – 1.5*	1	(5.6 %)	0	(0.0 %)	0.244^c^

a The proportion represents the percentage of patients with <16 nM at steady state per group.

b Chi-squared test.

c Fisher's exact test.

### 3.4 Model performance

**Fig. 2:**
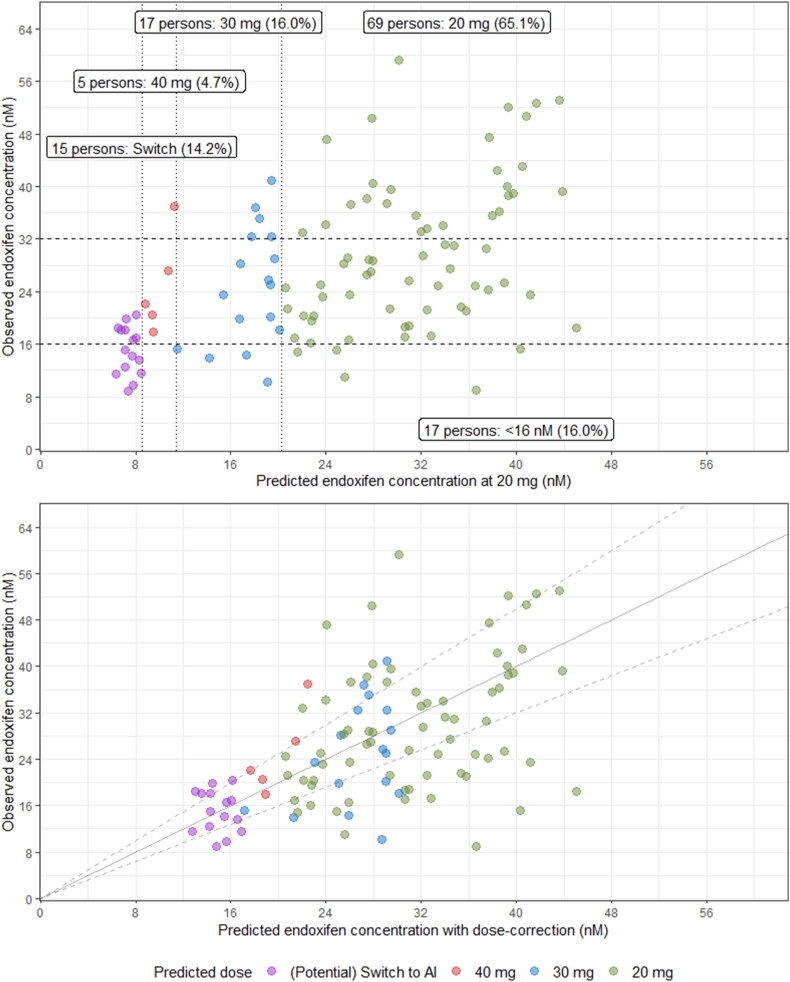
Distribution of steady-state endoxifen plasma concentrations in the intervention cohort. The top plot shows the distribution with the predicted endoxifen concentration when treated with 20 mg whereas the bottom plot shows the same distribution with a dose-corrected model prediction. dotted vertical lines depict the dosing cut-off points. Dashed horizontal lines depict the therapeutic index. Diagonal grey lines depict the unity line (solid) and the 80 – 125 % interval (dashed).

The model performance is depicted in Fig. 2 and Table 2, Table 3. Both the relative bias and mean absolute prediction error (MAPE) are beneath the prespecified external evaluation thresholds (**Supplementary** **Section III**). The model slightly overpredicts the steady-state endoxifen concentrations within the intervention cohort. Upon stratification by dose, the MAPE remained relatively consistent whereas the relative bias showed variability. Especially the 40 mg group was underpredicted by the model, although this group comprised of a group of 5 patients.

**Table 3:** Metrics evaluating the predictive value of the model predictions informed with no PK-information (*a priori*), stratified per dose group, or with either one or both samples obtained prior to reaching steady state.

Predictions	MAPE (%)	Relative bias (%)^a^	RMSE (nM)	F_80__– 125 %_
A priori	21.19	2.99	9.72	47.17
20 mg	*22.25*	*6.44*	*11.01*	*47.83*
30 mg	*21.33*	*11.63*	*8.21*	*47.06*
40 mg	*20.27*	*−20.27*	*7.31*	*40.00*
*(potential) switch to AI*	*21.65*	*−5.41*	*3.89*	*46.67*

<14 days sample^b^	27.46	0.13	10.68	37.21
4–6 weeks sample^c^	14.97	−10.91	7.36	63.73
Both samples	17.47	−12.16	7.91	53.49

a Negative percentage represents underprediction of the model.

b Available in 43 patients.

c Available in 102 patients. MAPE: median absolute prediction error. RMSE: root mean squared error. F_80__– 125 %_: The amount of patients inside the 80–125 % prediction/observation ratio.

When focusing on the therapeutic interval, the model significantly increased the proportion of patients inside the therapeutic interval for patients with the model-defined CYP2D6 activity ≤0.3 (Table 2) [14]. Only one patient in this group had a steady-state endoxifen level of <16 nM, in case the patients predicted to need a switch were actually switched. This one patient was treated with 30 mg tamoxifen after the model predicted the patient to reach endoxifen levels 0.02 nM above the 40 mg threshold. Furthermore, patients with a CYP2D6 activity ranging between 0.3 and 0.75 also benefit from a model-informed dose, albeit without statistical significance. In patients harboring a more active CYP2D6 genotype (>0.75), the model did not perform better compared to the control group as these patients were already adequately dosed with the standard 20 mg dose. A more detailed definition and explanation of CYP2D6 activity presented in the **Supplementary (Section I)**.

Among patients predicted to fall short of adequate endoxifen plasma concentrations, a majority indeed failed to attain adequate endoxifen plasma concentrations (>16 nM). Those achieving adequate levels showed a median endoxifen level of 18.2 nM and a maximum of 20.4 nM. The model-informed doses led to overexposure in six patients, which all but one occurred in the group treated with 30 mg tamoxifen.

The predictive value of pre-steady-state endoxifen samples is shown in Table 3. As the model-development dataset did not contain pre-steady state PK samples, these samples could not be used to change dosing. A post-hoc analysis using the samples was used to impute the pre-steady state endoxifen levels in the model and was further discussed in **Supplementary (Section IV).**

Quality of life and tamoxifen-related side effects results were missing for one patient, assigned to the 20 mg dose group. No significant or clinically relevant differences (>4.4 points for endocrine symptoms and >6.98 points for HR-QOL) between baseline and after 3 months in side-effects or quality of life were observed overall or in any individual dosing group (Table 4). At patient-level, 25 out of 68 (37 %) patients had a clinically relevant increase in side-effects in the 20 mg dosing group. This applied to 6 out of 17 (35 %) patients in the 30 mg group and 8 out of 20 (40 %) in the 40 mg group.

**Table 4:** Difference in side-effects overall and in the different dosing groups. Each group shows the mean score at baseline on the left and mean score after three months on the right. ^b^ES-19 comprises of 19 questions about specific hormone therapy related side-effects. ^c^HR-QOL includes questions from 4 domains about quality-of-life. None of the differences shown were significant or clinically relevant. Higher scores equate less side effects or better quality of life.

	Baseline	3 months	Differences
Total intervention cohort (*n* = 105)			
HR-QOL	86.1	87.0	+0.9
ES19	64.8	61.3	−3.5
20 mg dose group (*n* = 68)			
HR-QOL	88.6	88.4	−0.2
ES19	66.7	62.8	−3.9
30 mg dose group (*n* = 17)			
HR-QOL	79.6	80.8	+1.2
ES19	59.5	57.0	−1.5
40 mg dose group (*n* = 20)			
HR-QOL	82.8	87.6	+4.8
ES19	62.8	59.8	−3.0

## 4 Discussion

This study aimed to implement MIPD for oncology practice. A fundamental study by Joerger et al. proved MIPD could play an important role in individualized therapy for oncology patients [15]. In this study, initial paclitaxel therapy for patients with non-small-cell lung cancer (NSCLC) in a palliative setting was dosed, adjusted to several patient characteristics. Subsequent doses were directed by neutropenia levels and previous-cycle paclitaxel exposure, therefore combining MIPD and TDM to improve patient outcomes, such as treatment side-effects and overall survival (OS) [15]. The PREDICTAM study focused on putting MIPD into practice, by replacing TDM, specifically in adjuvant treatment for patients with hormone sensitive breast cancer. In contrast to the pharmacodynamic endpoints of the study by Joerger et al., this study mainly centered around pharmacokinetic objectives, such as attaining target endoxifen concentrations.

Although the difference in patients with endoxifen levels >16 nM in the intervention cohort was not significantly different from the control cohort, MIPD did result in less undertreatment when separate dose groups were compared. The model adequately identified patients who were at risk of being undertreated and identified patients who were unable to reach the therapeutic interval. In the intervention cohort, more patients than expected had low CYP2D6 activity scores, likely explaining the lower-than-expected percentage of patients reaching endoxifen levels >16 nM. Notably, a significant proportion of these patients belonged to the 'potential' switch AI group. More than half of these patients did not reach the therapeutic window and those who did, were mostly within the error margin of the model of 18.6 %. When excluding these patients, 91.5 % of the remaining individuals achieved endoxifen levels exceeding 16 nM, more accurately showing the potential of MIPD. In a post-hoc analysis early endoxifen samples were imputed into the model, informing unexplained inter-individual variability in the conversion rate from tamoxifen to endoxifen (**Supplementary** **Section IV**). If this was used to adjust dosing, the primary endpoint showed a significant improvement in the proportion of patients achieving adequate endoxifen exposure.

MIPD showed promise compared to conventional one-size-fits-all dosing of 20 mg tamoxifen. For example, patients for whom a tamoxifen dose of 20 mg was predicted, were at a low risk of undertreatment (endoxifen <16 nM in 7 % of these patients in our study). In cases where non-adherence is not suspected, omitting TDM for this dose-group may be considered. Also, for patients who were predicted in the ‘potential switch to AI’ group more than 50 % did not reach endoxifen levels ≥16 nM despite being treated with the highest dose of tamoxifen (40 mg). Especially among postmenopausal patients, initiating treatment with an AI may offer a more efficacious alternative in such cases. However, for other dose-groups, TDM still remains critical to verify adequate steady-state endoxifen levels and adjust tamoxifen doses when necessary. Nonetheless, giving a model-informed starting dose increased the adequate exposure significantly in the 40 mg group when reaching steady-state endoxifen exposure. Overall, this tailored approach reduces the need for TDM samples. While this was not an aim of our study, this approach increases the efficiency of accurate tamoxifen treatment due to fewer hospital visits being needed for TDM in this specific dose group. Additionally, this method may also facilitate earlier attainment of on-treatment endoxifen levels for all dose groups or prompt consideration for commencing treatment with an AI.

The mean scores for tamoxifen-related symptoms (ES19) and HR-QOL in our study were 61.3 and 87.0 points after 3 months of tamoxifen, respectively. These scores were comparable, or even slightly better, than in previous studies where tamoxifen toxicity was assessed in larger groups of patients (ES19: 59–62 points; HR-QOL 79–83 points). This may be explained by the short period of tamoxifen treatment in our study [[16], [17], [18]]. No significant or clinically relevant changes in toxicity or quality of life were observed either overall or in any individual dosing study group. These results are in contrast with previous tamoxifen studies, that commonly reported tamoxifen-related toxicity [19,20]. An explanation for this could be that our study was performed in patients who recently started tamoxifen therapy and were still recovering from other anti-cancer therapies such as surgery, radiotherapy and chemotherapy. However, although sample sizes per dose group were small, these results also imply that prescribing a dose of 30 or 40 mg tamoxifen to selected patients using MIPD, instead of the usual 20 mg dose, does not increase the risk of self-reported side-effects in the first three months of treatment. Probably, this can be explained by the fact that endoxifen levels of these patients remain within normal limits, despite using higher dosages. Although this offers reassurance regarding the risk at increased toxicity when using MIPD, caution is still warranted to avoid administering higher doses than necessary, particularly considering late and more rare side effects such as venous thromboembolism or endometrial abnormalities [21,22].

Endoxifen plasma samples obtained prior to steady-state may add to the early detection of patients unable to reach adequate endoxifen levels. While samples collected within the first 21 days of treatment lack informative value, samples taken after 4–6 weeks add predictive value to the model. When imputed in the model, more than half of patients who could benefit from a dose increase at steady-state are identified. These samples were not used to adjust dosing as the model was not developed using pharmacokinetic samples prior to reaching steady-state and could therefore lead to incorrect dose adjustments. However, in post-hoc analyses the ability of the model to use these samples to correctly recommend dose adjustments is shown. Other benefits of obtaining a pre-steady-state sample is that it informs the treating physician of possible treatment non-adherence. By addressing adherence issues early on, patients can be educated about the critical importance of consistent treatment compliance. Even though the model-development dataset of the PK model did not incorporate samples taken prior to steady state, it adequately estimates empirical Bayes estimates (EBE) leading to an adequate prediction of steady-state. The underprediction after imputation of the samples in this model might result in more false positive cases of patients that might need a dose increase, although this was not the case in our cohort. However, although the results obtained show the predictive value of pre-steady-state samples, the popPK model should be updated with these samples to allow precise prediction of endoxifen concentrations and therefore allowing to change the tamoxifen dose prior to achieving steady-state.

To date, several tamoxifen population PK models have been developed [14,[23], [24], [25], [26], [27]], yet only two of these models have been externally validated, of which one is used in this research. Thus far, the model described in this research is the only model that has been prospectively validated. In the externally validated model by Klopp-Schulze et al., the researchers showed that 92.8 percent of a simulation cohort can reach the therapeutic window [26]. In this research, we show that implementing MIPD in a prospectively followed cohort, 91.5 % of patients reach the therapeutic window, when physicians adhere to the proposed switch to an AI. The similarity in this proportion indicates that implementing other models will probably not lead to a significant increase in patients reaching the therapeutic window compared to the presented model.

The presented study is not without limitations. Most notably, the differences in CYP2D6 activity in the intervention and control cohort affected the primary endpoint. Therefore, the primary endpoint does not fully reflect the ability of the model to predict an adequate dose for each individual patient. Although the number of patients being adequately exposed is a clinically relevant endpoint, being the most suitable comparison for TDM, it only partly captures the ability of the model to adequately predict endoxifen plasma levels and, subsequently, the correct tamoxifen dosage. Especially not when the most influential covariate is differently distributed in the intervention cohort compared to the control cohort. Nevertheless, numerical model evaluation techniques confirm adequate predictive performance, especially with pre-steady-state plasma samples informing EBEs. Naturally, evaluating the effect using clinical outcomes such as recurrence should be used when feasible. However, considering the relatively low recurrence rate of breast cancer, such a study would take multiple years and many more patients rendering it unfeasible. Additionally, therapy adherence was not regularly assessed in the study. Several patients had lower endoxifen plasma concentrations at steady-state compared to the pre-steady-state samples, which indicates that treatment adherence may have affected the results of the primary outcome. Lastly, in this study we used the endoxifen threshold of 16 nM, a threshold determined in a large retrospective cohort study [7]. This threshold has not been confirmed prospectively and there are studies that found lower efficacy limits for endoxifen efficacy (i.e. 9 and 14 nM) [28,29]. Moreover, in studies with patients with a high risk for breast cancer or a history of breast carcinoma-in-situ, low doses of tamoxifen (5–10 mg) were proven effective in preventing breast cancer [30,31]. However, an advantage of MIPD is that it can be applied for endoxifen thresholds of whatever desired value.

This MIPD implantation study is the first in the landscape of solid breast tumors to adjust tamoxifen doses before the start of treatment. Implementation of MIPD did not result in a significantly higher proportion of patients achieving endoxifen levels ≥16 nM compared to the one-dose-fits-all strategy. This can be explained by a larger proportion of patients with impaired CYP2D6 activity in the intervention cohort compared to the control cohort. Implementation of MIPD showed promise compared to one-size-fits-all dosing 20 mg tamoxifen, especially in a subgroup recommended to be treated with 40 mg. Particularly when the dose recommendation is informed with a pre-steady-state sample at 4–6 weeks after treatment start, MIPD may ensure swift attainment of adequate steady-state endoxifen plasma levels. Moving forward, refining the early sampling strategy and showing its predictive value across diverse patient populations will be pivotal in optimizing tamoxifen treatment outcomes.

## Earlier presentation

This work has not been presented previously.

## CRediT authorship contribution statement

**Ruben Y.M. van Nijnatten:** Writing – review & editing, Writing – original draft, Project administration, Methodology, Formal analysis, Conceptualization. **Sanne M. Buijs:** Writing – review & editing, Writing – original draft, Project administration, Methodology, Investigation, Conceptualization. **Bram C. Agema:** Writing – review & editing, Writing – original draft, Methodology, Investigation, Formal analysis, Conceptualization. **Raphaël M.J. Fischer:** Project administration, Investigation. **Inge Ghobadi Moghaddam-Helmantel:** Writing – review & editing, Resources, Methodology. **Caroline M.E. Contant:** Writing – review & editing, Project administration. **Felix E. de Jongh:** Writing – review & editing, Project administration. **Auke M.T. Huijben:** Writing – review & editing, Project administration. **Manon Kop:** Writing – review & editing, Project administration. **Annemieke van der Padt-Pruijsten:** Writing – review & editing, Project administration. **Hanneke J.M. Zuetenhorst:** Writing – review & editing, Project administration. **Ron H.N. van Schaik:** Writing – review & editing, Investigation, Conceptualization. **Birgit C.P. Koch:** Writing – review & editing, Methodology, Conceptualization. **A. Jager:** Writing – review & editing, Conceptualization. **Stijn L.W. Koolen:** Writing – review & editing, Supervision, Methodology, Investigation, Conceptualization. **Ron H.J. Mathijssen:** Writing – review & editing, Supervision, Project administration, Investigation, Conceptualization.

## Ethics approval

The PREDICTAM (PREDICtion of TAMoxifen) trial was conducted as an open-label, single-arm intervention study at the Erasmus MC Cancer Institute in Rotterdam, the Netherlands. It received approval from the institutional review board (MEC-2022-0437) and was registered in the U.S. National Library of Medicine (clinicaltrials.gov; NCT05525481). All patients provided written informed consent.

## Financial support

No funding was received for this work.

## Declaration of competing interest

The authors declared no competing interests for this work.
