# Supplementary material for: Implementation of model-informed precision dosing for tamoxifen therapy in patients with breast cancer: A prospective intervention study
Source: Breast. 2025 Jan 9;79:103880. doi: 10.1016/j.breast.2025.103880 (PMC11783121; doi:10.1016/j.breast.2025.103880)
Supplement: Multimedia component 1 [file mmc1.docx]

**SUPPLEMENTARY MATERIALS**

**Supplementary section I**

The development and details of the used popPK model have been presented in a previous article (1). In that research several predictors for steady-state endoxifen concentrations were identified, including age, body height, body mass index (BMI), and most importantly, continuous CYP2D6 activity, based on CYP2D6 genotype (eq.1). Individual activity scores for each allele can be found in Table S1 whereas significant covariate-PK relations have been presented in previous research (1). Other factors such as liver function were not proven to affect tamoxifen PK and were therefore not obtained.

${Activity}_{CYP2D6}=\left( \frac{{Activity}_{Allele1}+{Activity}_{Allele2}}{2} \right)^{0.606}$ (1)

The dosing predictions were confined to 20, 30 or 40 mg tamoxifen. The model predicted the endoxifen level for each patient under the assumption of using the standard tamoxifen dose of 20 mg. Dosing cut-off points for predicted endoxifen levels were determined using ROC-curves. Patients with predicted endoxifen levels surpassing 20.23 nM were prescribed tamoxifen 20 mg, while those with predicted levels between 11.40 nM and 20.23 nM received 30 mg, and those with predicted levels between 8.56 nM and 11.40 nM were given 40 mg. Additionally, for patients anticipated to have endoxifen levels below 8.56 nM while on 20 mg tamoxifen, it was expected that they would not reach the therapeutic threshold of 16 nM when using tamoxifen 40 mg. These patients might benefit more from adjuvant aromatase inhibitors (AI). To test this hypothesis, in this study, these patients were treated with tamoxifen 40 mg. This subgroup of patients was defined as ‘(potential) switch to AI’ group.

**Supplementary section II**

*Pharmacokinetic and pharmacogenetic analyses*

A validated liquid chromatography-tandem mass spectrometry method (UPLC-MS/MS) was used for the quantification of all tamoxifen and endoxifen measurements (2). Blood withdrawals were performed at the moment of trough levels of tamoxifen and endoxifen.

*CYP2D6* genotyping was performed using the Infiniti (Autogenomics; Carlsblad, CA, USA) and the Quantstudio machines (ThermoFisher Scientific; Waltham, MA, USA) at the Clinical Chemistry laboratory of the Erasmus University Medical Center. The patients’ blood samples were assayed on the following genetic variants: *2-10, *12, *13, *14, *17, *29, and *41. When the activity of specific CYP2D6 alleles was unknown, the website (pharmvar.org/gene/CYP2D6) was consulted. The literature was searched for its activity and a comparable allele in the model was filled in.

**Supplementary section III**

To elucidate the potential of the POP-PK model, the patient-specific predicted endoxifen levels were compared with the observed steady-state endoxifen levels by quantifying the mean absolute prediction error (MAPE), relative bias and the root mean squared error (RMSE). A model is generally seen as potent when the relative bias is < 20% and the MAPE is < 35% and more than 30% of all prediction errors is within 80 – 125% limit. (3) Moreover, the POP-PK model's efficacy in identifying patients failing to achieve the 16 nM endoxifen threshold despite receiving the maximum tamoxifen dose of 40 mg, i.e. the ‘(potential) switch to AI’ group, was evaluated. Additionally, it was evaluated whether the model informed dose resulted in overexposure, which was defined as surpassing an endoxifen level of 32 nM while treated with a model-initiated dose increase. Both secondary endpoints were analyzed descriptively.

The predictive efficacy of early endoxifen levels (measured within 14 days or between 4-6 weeks after the initiation of tamoxifen treatment) was assessed in a post-hoc analysis by integrating them into the predictive model. The early endoxifen levels were not used to change tamoxifen doses during the study. Subsequently, the MAPE, relative bias, and RMSE were computed to evaluate the accuracy of predictions based on these early samples. Furthermore, an exploration was conducted to assess the potential impact of possessing this information, particularly in the context of acting if the early samples indicated levels below a certain threshold.

To investigate whether dose adjusting according to MIPD would lead to more toxicity, the difference in tamoxifen-related side effects and HR-QOL between start of therapy and after reaching steady-state endoxifen plasma levels was evaluated, stratified on separate dosing categories. The difference in tamoxifen-related side effects and quality of life between baseline and 3 months of therapy was compared using a paired sample t-test or a Wilcoxon signed-rank test when appropriate. Changes in ES or HR-QOL were seen as clinically relevant when they surpassed 0.5 of standard deviation at baseline. (4) The percentage of patients with clinically relevant changes in side effects per dose group were analyzed descriptively.

**Supplementary section IV**

During the study endoxifen samples prior to steady-state were obtained at the time of inclusions (<14 days of tamoxifen treatment, n=43) and after 4 – 6 weeks of treatment (n=102). Distribution of samples over time is provided in **Figure S1**. These samples were obtained to assess the predictive value of these samples for predicting steady-state concentrations. As the used model was not developed using pre-steady-state samples these were not used to affect the tamoxifen dose during treatment. In this post-hoc analysis, the samples were imputed in the popPK model used in this research (PMID: 36753957). A sample at the start of treatment (<14 days) deteriorated the predictive value of the model compared to the predictions prior to the start of treatment. Conversely, samples obtained between 4 – 6 weeks of treatment improved model predictions, although it introduced underprediction. Model evaluation by a visual predictive check, showed slight overprediction for the pre-steady-state samples, explaining the underprediction at steady-state (**Figure S2**). When imputing both pre-steady-state samples in the model, this did not improve the model predictions compared to only the sample taken after 4 – 6 weeks.

Remarkably, in 13.2% of patients, the endoxifen plasma concentration decreased at steady-state endoxifen concentrations compared to the 4-6 week sample. In more than half of these patients the endoxifen plasma concentration dropped over 10%. For two patients in the 20 mg group, this meant that their endoxifen level dropped under the critical 16 nM threshold at steady-state.

When using the early samples obtained at 4 – 6 weeks after treatment, 20 patients were expected to not reach endoxifen levels ≥16 nM at steady state. Fourteen out of fifteen patients identified as necessitating a switch to an AI fell within this group. Six of these patients will eventually achieve adequate endoxifen plasma levels (range 16.6 – 20.5 nM). The six other patients were treated with 40 mg (1 patient), 30 mg (4 patients) or 20 mg (1 patient). None of the five patients in this group that were treated with less than the maximum registered dose of 40 mg reached an endoxifen level >16 nM. Therefore, these patients would have profited from dose escalation. Four out of these five patients would have achieved adequate exposure with dose escalation toward 40 mg, assuming therapy compliance would remain similar. The addition of samples obtained between 4-6 weeks after treatment initiation did not identify any false positives that would have been unrightfully dose increased. Alternatively, it did not recognize three patients treated with 20 mg that did not reach ≥16 nM levels at steady state. These patients had 4 – 6 weeks endoxifen levels of 15.7, 16.4, 16.6 nM (**Table S2)**. For one patient who did not reach 16 nM treated with 20 mg, no pre-steady-state sample was available. If the early samples were used to change dose predictions, this would have changed the primary outcome. In that case 87.7% of the population would have achieved adequate endoxifen levels corresponding with a X^2^ = 5.15; *p* = 0.023.

**Table S1. Overview of the activity levels of the different *CYP2D6***

| *CYP2D6* allel | Activity level in POP-PK model |
| --- | --- |
| *1 | 1 |
| *2 | 0.560 |
| *3 | 0.066 |
| *4 | 0.047 |
| *5 | 0.040 |
| *6 | 0 |
| *7 | 0 |
| *9 | 0.378 |
| *10 | 0.103 |
| *17 | 0.156 |
| *29 | 0.490 |
| *31 | 0 |
| *41 | 0.110 |
| *1/*2 duplicate | 1.400 |

**Table S2. Overview of patients not reaching endoxifen levels ≥ 16 nM at steady-state**

| CYP2D6 allel 1 | CYP2D6 allel 2 | CYP2D6 activity score | Weight (kg) | Height (cm) | BMI (kg/m^2^) | Age (years) | Predicted endoxifen with 20 mg tamoxifen (nM) | Predicted dose (mg) | (Potential) switch to AI’ (Yes/No) | Predicted endoxifen level at predicted dose (nM) | Observed endoxifen level (nM) | Observed tamoxifen level (nM) | Predicted endoxifen after pre-SS sample | Pre-SS sample |
| --- | --- | --- | --- | --- | --- | --- | --- | --- | --- | --- | --- | --- | --- | --- |
| *4 | *4 | 0.157 | 106.6 | 181 | 32.54 | 57 | 6.38 | 40 | Yes | 12.76 | 11.5 | 866 | 8.81 | 6.22 |
| *4 | *4 | 0.157 | 73.8 | 172 | 24.95 | 48 | 7.10 | 40 | Yes | 14.2 | 12.5 | 376 | 9.64 | 8.40 |
| *4 | *7 | 0.103 | 53 | 158 | 21.23 | 62 | 7.13 | 40 | Yes | 14.26 | 15.1 | 433 | 11.2 | 11.6 |
| *4 | *4 | 0.157 | 76.7 | 160.5 | 29.77 | 50 | 7.38 | 40 | Yes | 14.76 | 8.89 | 596 | 9.44 | 5.05 |
| *4 | *4 | 0.157 | 73.4 | 175 | 23.97 | 60 | 7.71 | 40 | Yes | 15.42 | 14.2 | 666 | 13.8 | 11.9 |
| *6 | *41 | 0.172 | 100.1 | 170 | 34.64 | 67 | 7.82 | 40 | Yes | 15.64 | 9.76 | 531 | 9.58 | 5.03 |
| *4 | *41 | 0.214 | 109.5 | 173 | 36.59 | 58 | 8.29 | 40 | Yes | 16.58 | 13.6 | 422 | 9.92 | 6.82 |
| *4 | *41 | 0.214 | 116.5 | 161 | 44.94 | 57 | 8.43 | 40 | Yes | 16.86 | 11.6 | 319 | 7.99 | 6.37 |
| *41 | *41 | 0.262 | 82.1 | 165 | 30.16 | 60 | 11.45 | 30 | No | 17.175 | 15.2 | 434 | 13.4 | 12.5 |
| *2 | *4 | 0.486 | 136 | 188 | 38.48 | 56 | 14.16 | 30 | No | 21.24 | 13.9 | 253 | 15.7 | 13.2 |
| *2 | *5 | 0.482 | 90.1 | 164 | 33.5 | 50 | 17.29 | 30 | No | 25.935 | 14.3 | 342 | 12.0 | 8.93 |
| *2 | *41 | 0.515 | 81.9 | 173 | 27.36 | 55 | 19.10 | 30 | No | 28.65 | 10.2 | 302 | 11.6 | 8.08 |
| *2 | *5 | 0.482 | 63 | 169.5 | 21.93 | 65 | 21.65 | 20 | No | 21.65 | 14.8 | 245 | 15.2 | 14.0 |
| *1 | *2 | 0.860 | 113 | 166.5 | 40.76 | 43 | 24.90 | 20 | No | 24.9 | 15.1 | 206 | 18.1 | 16.6 |
| *1 | *9 | 0.798 | 108.4 | 171.5 | 36.86 | 55 | 25.60 | 20 | No | 25.6 | 11.0 | 145 | 18.0 | 16.4 |
| *1 | *1 | 1.000 | 82.8 | 162 | 31.55 | 56 | 36.62 | 20 | No | 36.62 | 9.02 | 387 | - | - |
| *1 | *2 | 0.860 | 45.9 | 149.5 | 20.54 | 50 | 40.29 | 20 | No | 40.29 | 15.2 | 245 | 21.4 | 15.6 |

Pre-SS sample: sample obtained 4 – 6 weeks after treatment initiation

- : pre-SS sample was not obtained

**
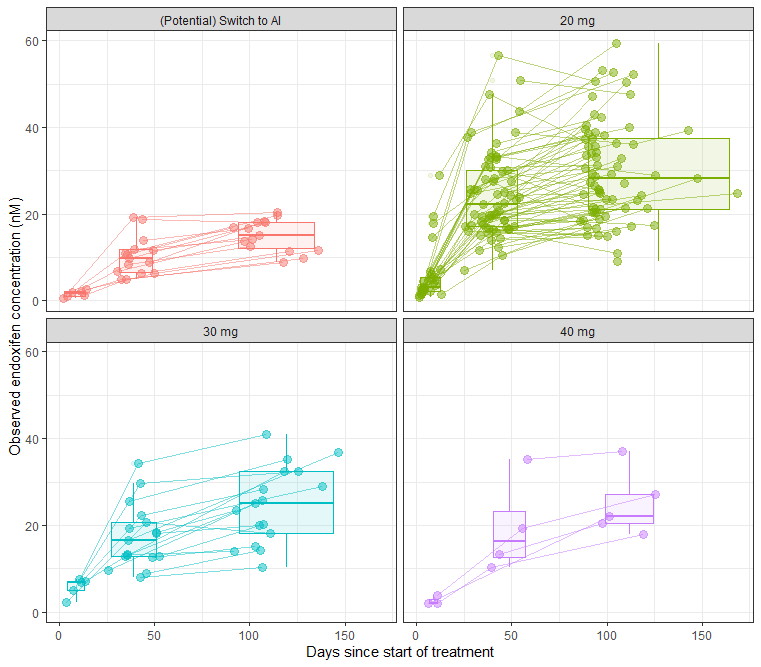
Figure S1. Distribution of all samples stratified on predicted dose group.**

**Figure S2. Visual Predictive Check for endoxifen samples obtained in the study**
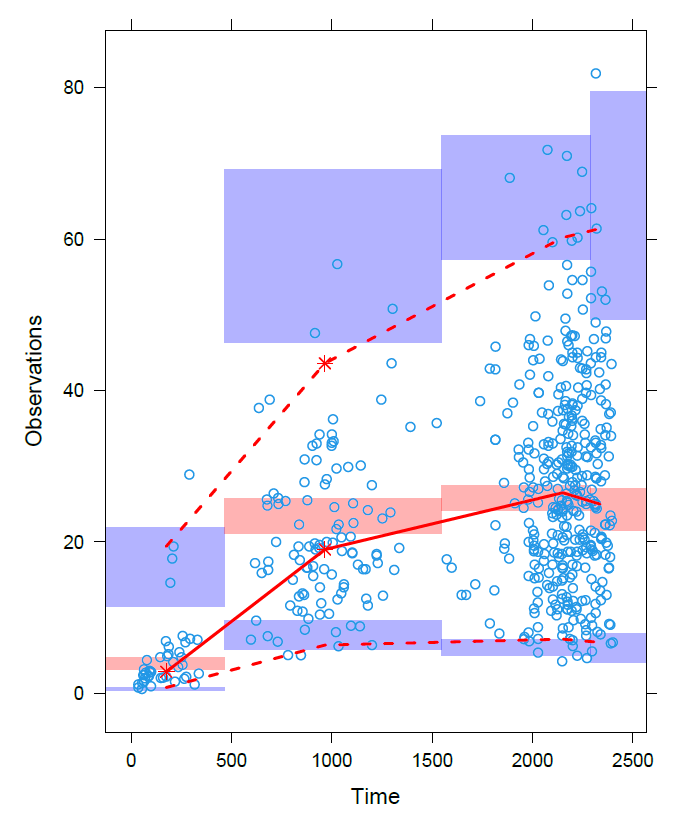


y-axis endoxifen nM, x-axis time in hours

1. Agema BC, Buijs SM, Sassen SDT, Mürdter TE, Schwab M, Koch BCP, et al. Toward model-informed precision dosing for tamoxifen: A population-pharmacokinetic model with a continuous CYP2D6 activity scale. Biomedicine & Pharmacotherapy. 2023;160:114369.

2. Binkhorst L, Mathijssen RH, Ghobadi Moghaddam-Helmantel IM, de Bruijn P, van Gelder T, Wiemer EA, Loos WJ. Quantification of tamoxifen and three of its phase-I metabolites in human plasma by liquid chromatography/triple-quadrupole mass spectrometry. J Pharm Biomed Anal. 2011;56(5):1016-23.

3. Zhao C-Y, Jiao Z, Mao J-J, Qiu X-Y. External evaluation of published population pharmacokinetic models of tacrolimus in adult renal transplant recipients. Br J Clin Pharmacol. 2016;81(5):891-907.

4. Fallowfield LJ, Leaity SK, Howell A, Benson S, Cella D. Assessment of quality of life in women undergoing hormonal therapy for breast cancer: validation of an endocrine symptom subscale for the FACT-B. Breast Cancer Research and Treatment. 1999;55(2):189-99.
